# Post-Translational Modification Networks of Contractile and Cellular Stress Response Proteins in Bladder Ischemia

**DOI:** 10.3390/cells10051031

**Published:** 2021-04-27

**Authors:** Jing-Hua Yang, Han-Pil Choi, Annie Yang, Roya Azad, Fengmei Chen, Zhangsuo Liu, Kazem M. Azadzoi

**Affiliations:** 1 Department of Surgery, VA Boston Healthcare System and Boston University School of Medicine, Boston, MA 02130, USA; jyangva@gmail.com; 2 Proteomics Laboratory, VA Boston Healthcare System, Boston, MA 02130, USA; han-pil.choi@va.gov; 3 Departments of Urology and Pathology, VA Boston Healthcare System and Boston University School of Medicine, Boston, MA 02130, USA; roia27@hotmail.com (R.A.); fccchenfm@zzu.edu.cn (F.C.); zhangsuoliu@zzu.edu.cn (Z.L.)

**Keywords:** ischemia, bladder, contractile proteins, cellular stress response, protein modification, amino acid substitution, amino acid polymorphism

## Abstract

Molecular mechanisms underlying bladder dysfunction in ischemia, particularly at the protein and protein modification levels and downstream pathways, remain largely unknown. Here we describe a comparison of protein sequence variations in the ischemic and normal bladder tissues by measuring the mass differences of the coding amino acids and actual residues crossing the proteome. A large number of nonzero delta masses (11,056) were detected, spanning over 1295 protein residues. Clustering analysis identified 12 delta mass clusters that were significantly dysregulated, involving 30 upregulated (R^2^ > 0.5, ratio > 2, *p* < 0.05) and 33 downregulated (R^2^ > 0.5, ratio < −2, *p* < 0.05) proteins in bladder ischemia. These protein residues had different mass weights from those of the standard coding amino acids, suggesting the formation of non-coded amino acid (ncAA) residues in bladder ischemia. Pathway, gene ontology, and protein–protein interaction network analyses of these ischemia-associated delta-mass containing proteins indicated that ischemia provoked several amino acid variations, potentially post-translational modifications, in the contractile proteins and stress response molecules in the bladder. Accumulation of ncAAs may be a novel biomarker of smooth muscle dysfunction, with diagnostic potential for bladder dysfunction. Our data suggest that systematic assessment of global protein modifications may be crucial to the characterization of ischemic conditions in general and the pathomechanism of bladder dysfunction in ischemia.

## 1. Introduction

Chronic bladder ischemia is a condition resulting from interruption of blood flow to the bladder due to arterial occlusive disorders [1,2,3]. Growing evidence from clinical and basic research suggest that ischemia is a key factor in the pathogenesis of bladder dysfunction [4,5,6]. Reduction of oxygen and nutrients in ischemia was shown to compromise bladder structure and lead to involuntary smooth muscle contractions, inflammation and fibrosis [7,8]. Although the incidence of ischemia in bladder dysfunction is well documented, the mediating molecules and downstream pathways contributing to structural damage and overactive contractions in bladder ischemia remain largely unknown. Ischemia impairs cellular energy homeostasis and provokes cellular stress [9,10]. Upregulation of cellular stress response molecules activates downstream pathways to signal energy deprivation and hypoxia and promote cell survival [11,12]. Cells use a variety of stress sensors to sense cellular stress conditions [13,14]. Upon sensing stress, cells activate survival signaling pathways to initiate defensive responses and protect cellular integrity [13,14]. The cellular stress response involves homeostatic mechanisms that regulate cells to signal stressors eliciting cell danger, such as hypoxia and energy deprivation [15,16]. We have previously reported that bladder ischemia inhibits the DNA-binding activity of a key antioxidant molecule, namely, nuclear factor-erythroid 2-related factor 2 (Nrf2), leading to the upregulation of mitochondrial stress protein mtHsp70 or glucose-regulated protein 75 (GRP75) and activation of the cellular stress response via heat shock protein 70 (Hsp70) [17].

Post-translational modifications (PTMs) are the processes of adding or removing modified groups to one or more amino acid residues on a translated protein for the modification of covalent processing [18]. Well-characterized PTMs include phosphorylation, acetylation, methylation, ubiquitination, glycosylation and succinylation. Depending on the domain and motif, PTMs can regulate cell function or compromise cellular functional and structural integrity and lead to the development of pathological conditions. Phosphorylation, for example, is a frequently occurring preposition in various processes of life activities. The phosphorylation of the Na^+^/K^+^-ATPase pump on the cell membrane controls the intracellular concentration of sodium and potassium ions [19]. Acetylation can dramatically change the function of a protein by changing its properties, which may influence protein conformation and interactions with substrates, cofactors and other macromolecules. For example, histone acetylation, occurring through the enzymatic addition of an acetyl group from acetyl coenzyme A to the epsilon-amino group of lysine residues in histones, is involved in the regulation of many cellular processes, including chromatin dynamics and transcription, DNA replication and DNA repair [20]. The PTMs of histone proteins, which include acetylation, phosphorylation, methylation and ubiquitination, can affect gene expression by changing chromatin structure or through the recruitment of histone modifiers. For methylation, the original structural sequence of peptide chains is changed after the methylation of proteins, and more information can be coded to regulate the interactions among the signaling molecules and between the signal molecules and target proteins [21]. By regulating such changes and coding, methylation contributes to numerous cellular functions or dysfunction by activating downstream mechanistic processes [21]. Protein ubiquitination is another common form of PTM that regulates a wide variety of ubiquitin protein substrates involved in cell function, as well as signaling pathways stimulated by pathological conditions [22]. Ubiquitination-directed modifications can mark proteins for degradation by the proteasome, compromise their cellular location, alter their activity and initiate or block protein interactions [22]. 

Recent studies have suggested that ischemia may be a key factor in provoking post-translational protein modifications in a variety of cells [23,24]. The mechanism appears to involve noxious free radicals in the ischemic cells that initiate modifications of the translated protein by adding or removing modified groups to the amino acid residues [25]. It was shown that the accumulation of reactive oxygen and nitrogen species in the ischemic tissues modifies the fibrinogen molecule and leads to inflammatory responses [26,27]. Analysis of retinal ischemia/reperfusion injury has revealed that DNA damage associated with histone PTMs may be a key contributing factor in neurovascular degeneration [28]. It was shown that transient cerebral ischemia activates various post-translational protein modifications involved in important neuronal functions and signaling pathways [29]. The mechanism appeared to involve ubiquitin aggregates, small ubiquitin-like modifier conjugation and ISGylation mediated by a ubiquitin-like protein, namely, Interferon-stimulated gene 15 (ISG15) [29]. The deacetylation of specific mitochondrial proteins by sirtuin preserves mitochondrial function and attenuates myocardial oxidative damage during ischemia/reperfusion [30]. Growing evidence suggests that the pathogenesis of ischemia/reperfusion injury is closely related to post-translational protein modifications at functional domains [28,30].

It is now well established from a variety of studies that the physical and chemical properties of proteins dictate cell function and regulate physiological functioning in living organisms. One of the main routes of proteome expansion is through protein modifications by various modification mechanisms, known or unknown, that are crucial to molecular reactions and functional pathways, as well as pathophysiological development in disease conditions [18,19,21,22]. Because of the constant addition of new PTMs, the proteome of an organ at any given moment in life is up to two or three times more complex than the encoding genomes would predict [31]. Certain limitations in protein sequencing technologies have imposed major challenges and made it difficult to determine global protein modifications throughout the proteome. Therefore, it remains virtually unknown how many different modifications are involved in tissue ischemia and which underlying pathways are activated after protein modifications by ischemia. As each modification is likely to provoke structural reactions with functional consequences, the systematic characterization of protein modifications may be a novel approach to define molecular mechanisms and downstream pathways of bladder dysfunction in ischemia. 

In the present study, mass spectrometry-based proteomic technologies were used to identify and systematically analyze protein modifications in bladder ischemia versus controls. Taking advantages of the high accuracy and rapid data acquisition of modern mass spectrometry technology, along with a modified shotgun proteomics method, all possible mass differences between coded amino acids and actual protein residues were analyzed. We report 23 different modifications of the rat bladder proteome in ischemia, spanning nearly 1300 modified protein sites. Our study reveals a large number of unreported modifications in the bladder that are polymorphous and discriminative under the ischemic condition. Differences in non-coded amino acids in bladder proteomics and changes in these protein pathways imply bladder sensitivity to ischemia and a close association between protein modifications and cellular stress responses to ischemia. Ischemia-regulated alterations of the bladder proteome may provide new insights into the molecular mechanisms and downstream pathways underlying bladder dysfunction.

## 2. Materials and Methods

### 2.1. Bladder Ischemia Model

Animal care and experimental protocols were in accordance with the guidelines of our Institutional Animal Care and Use Committee. Adult male apolipoprotein E knockout (ApoE^−/−^) rats (Envigo, Indianapolis, IN, USA) were used. The ApoE^−/−^ rat is a widely accepted model for studies of arterial atherosclerosis as it displays spontaneous hypercholesterolemia. To promote arterial plaque formation and expedite atherosclerotic occlusive disease in the ApoE^−/−^ rats, the endothelial layer of the iliac arteries was partially denuded using a 2F Fogarty arterial embolectomy catheter (Baxter Healthcare Corporation, Dixon, CA, USA), as previously described [1,2,3,7,8]. In brief, under general anesthesia with continuous inhalation of 1–2% isoflurane mixed with oxygen, a Fogarty catheter was inserted to the right and left femoral arteries and advanced to the abdominal aorta. After this, the balloon of the catheter was inflated and subsequently withdrawn to the respective femoral artery while rotating the catheter. To achieve endothelial denudation, arterial ballooning was repeated five times on each side. The sham control group underwent similar procedures without arterial ballooning. After 8 weeks, the degrees of ischemia were determined by measuring blood flow at five different bladder sites using laser Doppler blood flowmetry, as we have previously reported [1,2,3,7,8]. We sacrificed the rats at 8 weeks after arterial ballooning because ApoE^−/−^ rats develop significant arterial occlusive disease and bladder ischemia at this time point. 

### 2.2. Sample Preparation for Proteomic Analysis 

Detrusor tissue samples containing contractile smooth muscle were collected from three animals with severe bladder ischemia and three sham control bladders and were processed for proteomic analysis. Bladder tissue samples were washed separately three times with ice-cold PBS (0.1 M Na_2_HPO_4_, 0.15 M NaCl, pH 7.5) and re-suspended in 1 mL of chilled 1 × RIPA lysis buffer (Millipore 20-188, MilliporeSigma, Burlington, MA, USA) supplemented with 10 μL of 500 mM DTT and 10 μL of protease inhibitor cocktail (MilliporeSigma, Burlington, MA, USA) to protect against oxidation and degradation. After being sonicated three times for 5 s on ice, lysates were centrifuged at 13,000× *g* for 20 min at 4 °C. Supernatants were collected and protein concentrations were determined using the bicinchoninic acid (BCA) assay (MilliporeSigma, Burlington, MA, USA).

### 2.3. Sodium Dodecyl Sulfate Polyacrylamide Gel Electrophoresis (SDS-PAGE) Fractionation and Trypsin Digestion

One hundred micrograms of each bladder tissue lysate protein were run into each well on the 15% SDS-PAGE gel separately, stained with Coomassie Brilliant Blue, and divided into 10 fractions based on molecular weight. Gel slices were washed with 50 mM ammonium bicarbonate, dehydrated with acetonitrile, and lyophilized in a SpeedVac (Thermo Scientific, Waltham, MA, USA). Proteins were digested, in-gel, with sequencing-grade modified trypsin (Promega, Madison, WI, USA) at a protein:trypsin ratio of 25:1 in 50 mM ammonium bicarbonate (pH 8) overnight at 37 °C. The extracted peptides from each gel slice were desalted by ZipTip C18 columns (MilliporeSigma, Burlington, MA, USA) and dried in a SpeedVac for LC-MS/MS analysis. The RAW data of 10 fractions from each tissue sample were searched against the UniProt database as a group.

### 2.4. Liquid Chromatography–Tandem Mass Spectrometry (LC-MS/MS) Analysis 

Desalted peptides were subjected to peptide fractionation by means of liquid chromatography on an EASY-nLC 1000 system (Thermo Scientific, Waltham, MA, USA). Samples were trapped on a C18 pre-column and fractionated with a long C18 column (300 × Ø 0.075 mm, 3 μm; Reprosil, Germany) with a 180-min linear gradient of 5–35% acetonitrile/0.1% formic acid at a flow rate of 250 nL/min (solvent A, 0.1% formic acid in water; solvent B, 0.1% formic acid in acetonitrile). MS and MS/MS spectra were acquired using a LTQ-Orbitrap Elite mass spectrometer (Thermo Scientific, Waltham, MA, USA) in a data-dependent mode. One biological sample was repeated 3 times on the LC-MS/MS and the delta masses were open-searched individually. The delta masses from all samples were clustered and the average of 3 repeats were given. The spray voltage was 2.1 kV and the capillary temperature was 275 °C. MS spectra were acquired in profile mode in the *m/z* range of 350−1800 at a resolution of 60,000 at 400 *m/z*. The automatic gain control was 1 × 10^6^. MS/MS fragmentation of the 30 most intense peaks was performed for every full MS scan in the collision-induced dissociation mode. Normalized collision energies were set to 35% with an activation time of 10 ms for the MS/MS method. The maximum precursor ion injection time for MS/MS was 100 ms. The repeat count was set to 2, and the dynamic exclusion duration was 90 s. The minimal signal required for MS/MS was set at 1000. 

### 2.5. The Clustered Delta Masses 

Typically, MS/MS spectra were searched against the rat protein database using the wildcard search in Byonic software [32] and filtered with Score ≥ 300, DeltaModsScore ≥ 10, FDR2D ≤ 0.01 and FDR_uniq.2D ≤ 0.01. All amino acid residues that were different from coded genomic sequences were identified to calculate nonzero delta masses (delta mass > 0.01), the mass differences between the actual and theoretical (coded) amino acids. The amino acids which had nonzero delta masses were designated as “non-coded amino acids (ncAAs)”. For occurrence analysis, delta masses were rounded to four decimal places and the number of redundancies was considered as the frequency of the delta mass. For clustering, delta masses were divided into subgroups with 1-Da intervals, bounded by *n* − 0.5 and *n* + 0.5 Da (*n* = −200 to 1000). Delta masses in each mass window were analyzed by means of multivariate clustering using Gaussian mixture components [33] with the following constraints: (1) peak half-width > 1 ppm; (2) peak distance > 2 peak widths; (3) cluster size >20. Clusters within each window were determined using the Bayesian information criterion (BIC) [34], in which larger BIC values indicate a stronger model and confidence in the number of clusters. Clusters in each window were fitted individually with Gaussian regression to calculate the peak value (clustered delta mass), the standard deviation (SD), and the goodness-of-fit (R^2^). Clustered delta masses were annotated with previously known PTMs and amino acid substitutions in the UniMod, RESID, ExPASy and ABRF databases. Matched clusters were considered as the true delta masses in the examined protein samples. Unmatched clusters were assigned with confidence only when the goodness-of-fit R^2^ > 0.5 and/or the cluster was predominantly associated at a single amino acid (>50%). The significance of the delta masses (modifications) was defined by parameters of a Gaussian distribution, assuming the errors were random. Thus, a significant delta mass was defined with R^2^ > 0.5, which is a very high standard for non-linear regression. While comparing 3 ischemia versus 3 controls, only a ratio > 2-fold and *p* < 0.05 between the ischemia and control group were considered “significant”.

### 2.6. Gene Ontology, Protein–Protein Interaction Network and Pathway Analysis

The gene ontology enrichment analysis of ischemia-associated ncAA-containing proteins was performed using the PANTHER (Protein ANalysis THrough Evolutionary Relationships) Classification System (Version 15.0, released 14 February 2020) (http://pantherdb.org/). The protein–protein interaction network for the ischemia-associated ncAA-containing proteins was annotated using the STRING (Search Tool for Recurring Instances of Neighbouring Genes) database (Version 11.0) (http://string-db.org/). For pathway analysis of the ischemia-associated ncAA-containing proteins, the DAVID (The Database for Annotation, Visualization and Integrated Discovery) 6.8 bioinformatics resource (https://david.ncifcrf.gov/) was used.

### 2.7. Statistical Analysis 

SPSS 19.0 software (IBM, Armonk, NY, USA) was used for statistical analysis, and groups were compared using two-tailed student’s *t*-tests. ANOVA was used to assess variation between the groups.

## 3. Results

### 3.1. Validation of Bladder Ischemia in the Rat Model 

Balloon endothelial denudation in the ApoE^−/−^ rats led to diffuse atherosclerotic disease in the iliac arteries with a significant decrease in bladder blood flow. Laser Doppler blood flowmetry revealed that bladder blood flow in the atherosclerotic rats was significantly diminished to 3.7 ± 0.6 mL/min/100 g tissue in comparison with 8.7 ± 1.2 mL/min/100 g tissue in sham controls (*p* = 0.007), suggesting bladder ischemia in the treated animals.

### 3.2. Delta Mass Clusters in the Bladder Proteome

The shotgun proteomics approach was modified to determine delta masses between the genetically coded amino acids and the actual protein residues in the bladder proteome. Briefly, total proteins were isolated from three ischemic and three sham control bladders and digested with trypsin in-gel. The peptides (MS) and their fragments (MS/MS) were repeatedly determined at least three times using liquid chromatography-tandem mass spectrometry (LC-MS/MS) to acquire high-quality spectrum data. By allowing any possible protein residues in peptides, the acquired MS and MS/MS data were matched against the genetically coded protein sequences using the multi-blind spectral alignment algorithm Byonic [32]. A large number of nonzero delta masses (11,056) were detected, spanning over 1295 protein residues and falling into 23 clusters with excellent Gaussian distributions (Table S1). By definition, these residues with clustered delta masses reflected the occurrence and progressive accumulation of non-coded amino acids (ncAAs) in the bladder proteome that emerged steadily in ischemia versus controls.

### 3.3. Ischemia-Regulated Amino Acid Substitutions in the Bladder Proteome

However, 12 delta mass clusters among 23 clusters were significantly dysregulated in the ischemic tissues (R^2^ > 0.5, ratio > 2-fold, *p* < 0.05), which were considered potential ischemia-regulated protein modifications (Table 1). 

Among 12 ischemia-associated delta mass clusters, seven apparently matched amino acid substitutions in accordance with their molecular weights. The most abundant delta mass cluster in ischemia was 14.0172 (*n* = 418), which matched amino acid substitutions (D > E, N > Q, G > A, S > T or V > I/L) with a mass error less than 0.002 Da. This delta mass was found at P02770@431V (ratio = −20.2, *p* = 0.0007, *n* = 415) and P02770@424E (ratio = 3.1, *p* = 0.0001, *n* = 3) of albumin-like protein (Table S2). Based on the comparison of the genomic DNA and cDNA databases, P02770@431V+14.0172 matched a missense point mutation from codon GTT to codon ATT, indicating a V > I/L substitution in the ischemic bladder tissues. In contrary, another delta mass at P02770@424E+14.0172 did not match any known point mutations at the genome level, although it matched a yet unreported post-translational modification to form a glutamic acid methyl ester at 424E of glycated tissue albumin.

Although several other ischemia-regulated delta mass clusters matched the molecular weights of amino acid substitutions, they appeared not to be generated by point mutations at the DNA and/or RNA levels. For instance, the delta mass cluster of −33.9858 matched amino acid substitution F > L/I or M > P. However, this delta mass was found at the cysteine residues on creatine kinase B-type (P07335@254C−33.9858 (ratio = −7.1, *p* = 0.008, *n* = 16)), myosin regulatory light polypeptide 9 (Q64122@109C−33.9858 (ratio = −5.1, *p* = 0.010, *n* = 5)), L-lactate dehydrogenase A chain (P04642@163C−33.9858 (ratio = −4.0, *p* = 0.014, *n* = 4)), and albumin-like protein (P02770@192C−33.9858 (ratio = 4.1, *p* = 0.014, *n* = 4) and P02770@99C−33.9858 (ratio = 4.3, *p* = 0.034, *n* = 4)) (Table S2). In fact, loss of this delta mass at cysteine could not make other standard amino acids. However, C-33.9858 matched the removal of H_2_S from cysteine that generated dehydroalanine [35], suggesting the production of hydrogen sulfide and dehydroalanine from cysteine in association with ischemia. Anther ischemia-associated delta mass cluster, 31.9913, could make P > E substitutions. However, it was found at P31232@86M+31.9913 (ratio = 5.2, *p* = 0.011, *n* = 5), a methionine residue of transgelin, likely to be generated by dioxidation (Table S2).

### 3.4. Pathway Analysis of the Ischemia-Associated ncAA-Containing Proteins

There were 30 proteins which contained upregulated ischemia-associated ncAAs (R^2^ > 0.5, ratio > 2, *p* < 0.05) and 33 proteins which contained downregulated ischemia-associated ncAAs (R^2^ > 0.5, ratio < −2, *p* < 0.05) (Table 2 and Table S3). To systemically analyze the functions and understand the underlying regulatory pathways of these ischemia-associated ncAA-containing proteins (R^2^ > 0.5, ratio > 2-fold, *p* < 0.05), we performed DAVID analysis in order to further illuminate the functional annotations of those proteins. The DAVID database contains an integrated biological knowledgebase and extracts biological meaning from large gene/protein lists systematically.

DAVID analysis showed that the ischemia-associated ncAA-containing proteins were involved in the following signal transduction and regulatory pathways (Table 3): (1) smooth muscle contraction; (2) scavenging of heme from plasma; (3) heat shock transcription factor (HSF)1-dependent transactivation; (4) tetrahydrobiopterin (BH4) synthesis; recycling, salvage and regulation; (5) attenuation phase; (6) erythrocytes taking up oxygen and releasing carbon dioxide; and (7) endothelial nitric oxide synthase (eNOS) activation. In the smooth muscle contraction pathway, most of the involved proteins belong to upregulated ischemia-associated ncAA-containing proteins. In contrast, most of the involved proteins in the pathway of HSF1-dependent transactivation belong to downregulated ischemia-associated ncAA-containing proteins.

**Figure 1:**
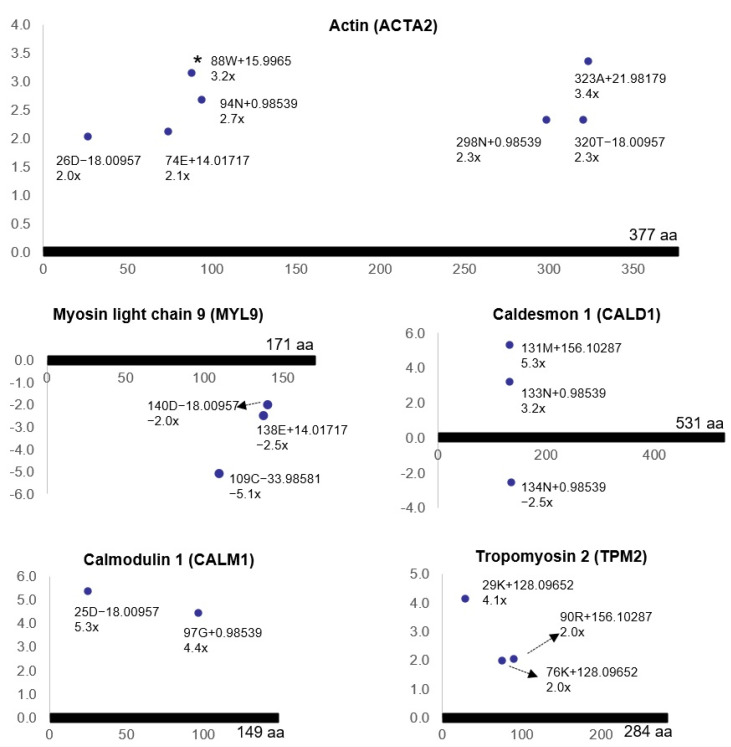
The modified residues of the proteins involved in smooth muscle contraction (R^2^ > 0.5, ratio > 2, *p* < 0.05). Each dot represents an ncAA of each protein. The annotation information of each dot is as follows: modified (protein) position, modified amino acid residue, delta mass and ratio of modification. The horizontal axis represents the amino acid position of each protein; The vertical axis represents the ratio of modification (up or down). The asterisk denotes the oxidation of actin. The possible modification for each nonzero delta mass is as follows: −18.00957: E > pyro-E, dehydration; 14.01717: methylation, D > E, N > Q, G > A, S > T, V > I/L; 15.9965: oxidation or hydroxylation, A > S, F > Y; 0.98539: deamidation, N > D, Q > E; 21.98179: sodium adduct; −33.98581: F > L/I, C > deHA, M > P; 156.10287: addition of arginine; 128.09652: addition of lysine.

Bladder ischemia resulted in differential (post-translational) modifications of the proteins involved in the smooth muscle contraction pathway, including contractile protein actin (ACTA2), myosin light chain 9 (MYL9), caldesmon 1 (CALD1), calmodulin 1 (CALM1) and tropomyosin 2 (TPM2) (Figure 1).

**Figure 2:**
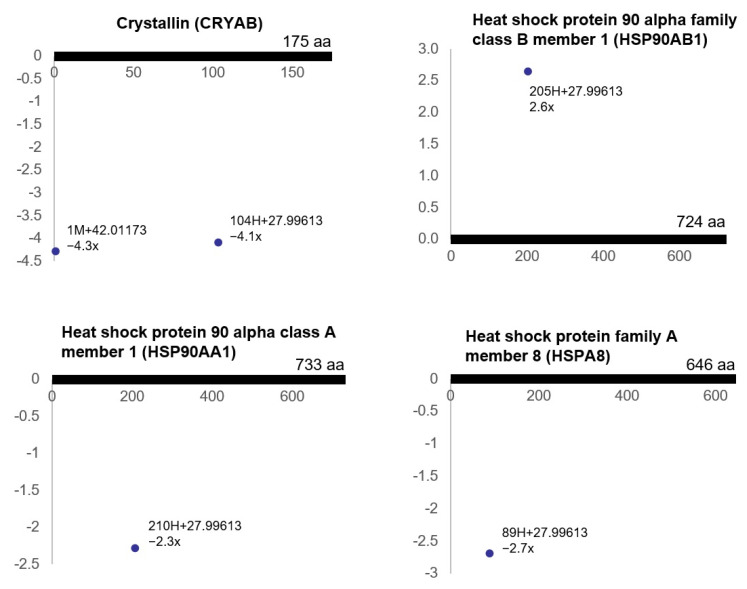
The modified residues of the proteins involved in HSF1-dependent transactivation (R^2^ > 0.5, ratio > 2, *p* < 0.05). Each dot represents an ncAA of each protein. The annotation information of each dot is as follows: modified (protein) position, modified amino acid residue, delta mass and ratio of modification. The horizontal axis represents the amino acid position of each protein; The vertical axis represents the ratio of modification (up or down). The possible modification for each nonzero delta mass is as follows: 42.01173: acetylation, S > E; 27.99613: formylation, S > D, T > E.

Marked changes in stress response molecules were also evident in the ischemic tissues, suggesting cellular stress in bladder ischemia. Ischemia provoked differential (post-translational) modifications of the proteins involved in the HSF1-dependent transactivation pathway, including crystallin (CRYAB), heat shock protein 90 alpha family class B member 1 (HSP90AB1), heat shock protein 90 alpha class A member 1 (HSP90AA1) and heat shock protein family A (HSP70) member 8 (HSPA8) (Figure 2).

**Figure 3:**
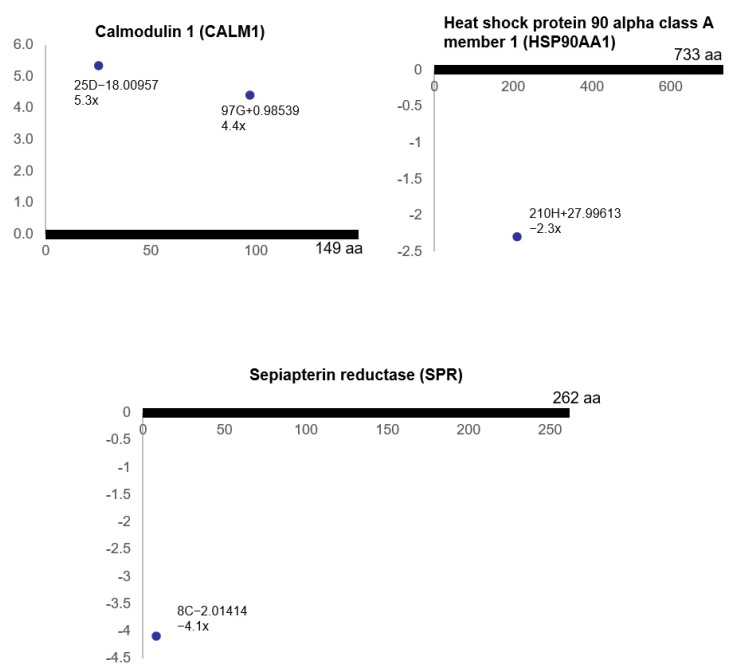
The modified residues of the proteins involved in tetrahydrobiopterin (BH4) synthesis, recycling, salvage and regulation (R^2^ > 0.5, ratio > 2, *p* < 0.05). Each dot represents ncAA of each protein. The annotation information of each dot is as follows: modified (protein) position, modified amino acid residue, delta mass and ratio of modification. The horizontal axis represents the amino acid position of each protein; the vertical axis represents the ratio of modification (up or down). The possible modification for each nonzero delta mass is as follows: −18.00957: E > pyro-E, dehydration; 0.98539: deamidation, N > D, Q > E; 27.99613: formylation, S > D, T > E; −2.01414: disulfidelation, V > P, −2H(didehydro).

Bladder ischemia also resulted in differential (post-translational) modifications of the proteins involved in the tetrahydrobiopterin (BH4) synthesis, recycling, salvage and regulation pathway, including calmodulin 1 (CALM1), heat shock protein 90 alpha class A member 1 (HSP90AA1) and sepiapterin reductase (SPR) (Figure 3).

### 3.5. Gene Ontology Analysis of the Ischemia-Associated ncAA-Containing Proteins

**Figure 4:**
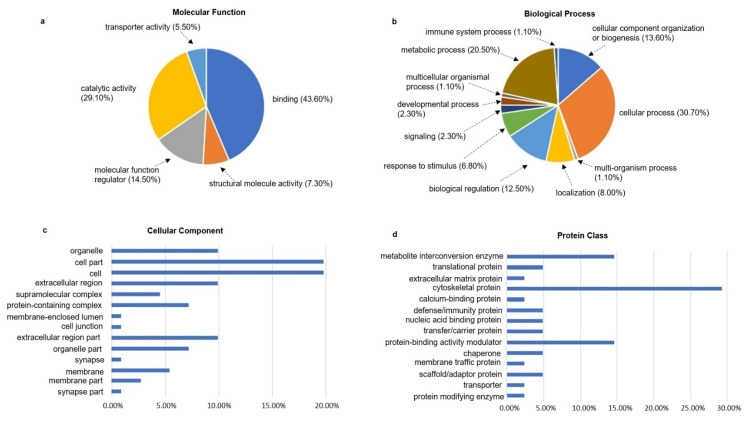
Gene ontology analysis of the ischemia-associated ncAA-containing proteins. The ischemia-associated ncAA-containing proteins (R^2^ > 0.5, ratio > 2-fold, *p* < 0.05) were categorized using the PANTHER database. (**a**) Molecular function; (**b**) biological process; (**c**) cellular component; (**d**) protein class. Each percentage number indicates the percentage of gene hits classified to each category out of the total number of molecular function/biological process/cellular component/protein class hits.

Cell components, molecular functions and biological processes are closely related. Cell components describe the location of a gene product in a cell. Molecular functions describe the molecular biological activity and function of a gene or gene product. Biological processes are usually composed of a variety of molecular functions in order to perform a certain biological behavior. The gene ontology (GO) of the ischemia-associated ncAA-containing proteins (R^2^ > 0.5, ratio > 2-fold, *p* < 0.05) was analyzed using the PANTHER database. The two most obvious changes in molecular function were binding (43.6%) and catalytic activity (29.1%), which were followed by molecular function regulator (14.5%), structural molecule activity (7.3%) and transporter activity (5.5%) (Figure 4a). With regards to the biological process, the ischemia-associated ncAA-containing proteins were mostly clustered into cellular process (30.7%) and metabolic process (20.5%). In addition, other changes in biological process in ischemia were identified in cellular component organization or biogenesis, biological regulation, localization, response to stimulus and other processes shown in Figure 4b. In terms of cellular components, the most significant changes in ischemia were found in the cell part (19.8%) and cell (19.8%). In addition, organelle (9.9%), extracellular region (9.9%) and extracellular region part (9.9%) were also significantly changed in ischemia (Figure 4c). To further comprehend the potential roles of the ischemia-associated ncAA-containing proteins, we also analyzed the protein classes of those proteins and found that they were mainly distributed among cytoskeletal proteins (29.3%), metabolite interconversion enzymes (14.6%) and protein-binding activity modulators (14.6%) (Figure 4d).

### 3.6. Protein–Protein Interaction Network Analysis of the Ischemia-Associated ncAA-Containing Proteins

**Figure 5:**
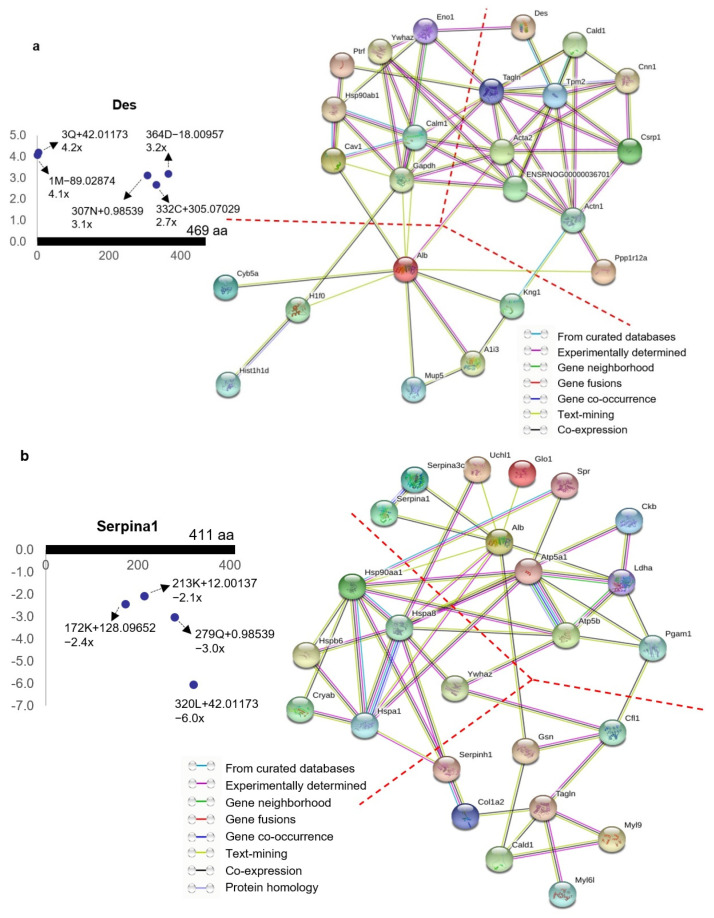
Protein–protein interaction networks of ischemia-associated ncAA-containing proteins. Network nodes and edges represent proteins and protein–protein associations, respectively. The color of the network edge indicates the type of interaction evidence. (**a**) Protein–protein interaction networks formed between 30 upregulated ischemia-associated ncAA-containing proteins (R^2^ > 0.5, ratio > 2, *p* < 0.05). Each protein had upregulated ischemia-associated ncAAs. Des is shown as an example. The annotation information of each dot, which represents ncAA, is as follows: modified (protein) position, modified amino acid residue, delta mass and ratio of modification. The horizontal axis represents the amino acid position of Des; the vertical axis represents the ratio of modification. The interaction networks were broadly divided into three clusters: cell signaling-related proteins (left), cytoskeleton-associated proteins (right), and binding/transport-related proteins (bottom) (24 connected proteins were shown and the clusters were divided with dotted lines). The possible modification for each nonzero delta mass is as follows: 42.01173: acetylation, S > E; −18.00957: E > pyro-E, dehydration; −89.02874: removal of initiator methionine from N-terminus, then acetylation of the new N-terminus; 0.98539: deamidation, N > D, Q > E; 305.07029: glutathione disulfide. (**b**) Protein–protein interaction networks formed between 33 downregulated ischemia-associated ncAA-containing proteins (R^2^ > 0.5, ratio < −2, *p* < 0.05). Each protein had downregulated ischemia-associated ncAAs. Serpina1 is shown as an example. The annotation information of each dot, which represents ncAA, is as follows: modified (protein) position, modified amino acid residue, delta mass and ratio of modification. The horizontal axis represents the amino acid position of Serpina1; the vertical axis represents the ratio of modification. The interaction networks were broadly divided into three clusters: molecular chaperones (left), metabolic enzymes (right), and cytoskeleton-associated proteins (bottom) (25 connected proteins were shown and the clusters were divided with dotted lines). The possible modification for each nonzero delta mass is as follows: 128.09652: addition of lysine; 12.00137: formaldehyde adduct; 0.98539: deamidation, N > D, Q > E; 42.01173: acetylation, S > E.

To further explore the potential interactions between the ischemia-associated ncAA-containing proteins (R^2^ > 0.5, ratio > 2-fold, *p* < 0.05), we used STRING, which is a database of known and predicted protein interactions, and these interactions include direct (physical) and indirect (functional) associations. STRING analysis of the ischemia-associated ncAA-containing proteins revealed that multiple interaction networks were formed between them. The protein–protein interactions of the 30 upregulated ischemia-associated ncAA-containing proteins (R^2^ > 0.5, ratio > 2, *p* < 0.05) based on the STRING analysis showed that the interaction networks were broadly divided into three clusters: cell signaling related proteins, cytoskeleton associated proteins and binding/transport related proteins (Figure 5a). STRING analysis of the 33 downregulated ischemia-associated ncAA-containing proteins (R^2^ > 0.5, ratio < −2, *p* < 0.05) indicated that the interaction networks formed between them were mainly divided into three clusters: molecular chaperones, metabolic enzymes and cytoskeleton associated proteins (Figure 5b). Among the molecular chaperone cluster, stress responsive heat shock proteins (HSPs) were predominant.

## 4. Discussion

Subcellular processes involved in protein synthesis, by themselves, do not directly result in the production of macromolecules with pertinent functional and structural properties. Many proteins must undergo one or more co-translational and/or post-translational modifications to activate downstream pathways and regulate cell function [36]. However, pathological modifications triggered by disease conditions can compromise protein structures, modify protein functional domains and disrupt the folding of proteins into defined three-dimensional structures. Adverse modifications and aberrant folding of proteins result in aggregation and lead to the accumulation of potentially toxic species in the cells. To protect against these negative outcomes, cells harbor a well-coordinated network of molecular chaperones to prevent cellular stress and promote cell function [37]. Pathological conditions such as ischemia expose the protein structure to a highly dynamic state that mandates constant molecular chaperone surveillance to ensure protein homeostasis. Analysis of post-translational protein modifications by proteomics may provide a novel approach for detecting regulatory molecules and downstream pathways involved in the pathogeneses of ischemic disorders following energy/oxygen deprivation in the affected tissues. Herein, we report a systematic workflow for identifying all possible protein residues, including those not directly encoded by their genomic sequences, namely, non-coded amino acids (ncAAs) in the proteome. By measuring the delta masses between actual protein residues and coded amino acids, over 11,000 non-zero delta masses were detected in the rat bladder and these non-zero delta masses were grouped into 23 clusters, where each cluster represented a specific chemical reaction occurring on the side chain of the modified proteins. There were 30 proteins which had upregulated ncAAs (R^2^ > 0.5, ratio > 2, *p* < 0.05) and 33 proteins which had downregulated ncAAs (R^2^ > 0.5, ratio < −2, *p* < 0.05) in bladder ischemia.

**Figure 6:**
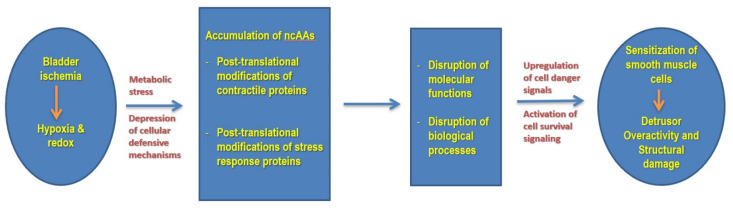
Graphical model of molecular and cellular consequences of post-translational modifications of contractile and stress response proteins in bladder ischemia. ncAAs: non-coded amino acids.

Our present study suggests that a lack of blood flow and subsequent decline in nutrients and oxygen levels compromise protein structure via post-translational modification mechanisms, leading to the accumulation of aberrant molecules and activation of downstream pathways in the ischemic cells [13]. The reactome pathway analysis of the ischemia-associated ncAA-containing proteins using DAVID indicated that bladder ischemia resulted in differential (post-translational) modifications of the proteins involved in the smooth muscle contraction pathway, including actin, myosin light chain 9, caldesmon 1, calmodulin 1 and tropomyosin 2. The actin cytoskeleton plays a key role in the establishment and maintenance of subcellular structures and cell function. We have previously reported that ischemia reduces the bladder’s antioxidant capacity, leading to the unconfined or inappropriate production of free radical species [38]. The accumulation of free radicals in bladder ischemia impairs subcellular elements through mechanisms involving DNA damage, protein oxidation and lipid peroxidation, leading to sequential structural damage in subcellular components including mitochondria, the endoplasmic reticulum, the nucleus and the cell membrane [7,8]. These changes activate cell danger signals and provoke degenerative responses, leading to the loss of smooth muscle cells and accumulation of connective tissue. We also reported that the ischemic bladder is associated with actin cytoskeleton signaling and aberrant smooth muscle contractile activity [1]. Assessment of ischemic bladder tissues in the organ bath showed significant smooth muscle hypersensitivity to both electrical field stimulation and carbachol [1,2,3]. Our present study revealed a significant increase in actin oxidation (3.2-fold) (Figure 1) that may contribute to increased smooth muscle contractile activity and detrusor overactivity in bladder ischemia. Oxidative modification of actin seemed to play a key role in the pathophysiology of ischemic disorders in rat hearts [39,40]. Oxidation of cysteines in actin is generally thought to slow the polymerization/elongation of G-actin [41,42] and make F-actin more fragile [43]. Furthermore, oxidation of methionines in actin by the molecule interacting with CasL (MICAL) promotes, in synergy with cofilin, the disassembly of actin filaments [44]. The consequences of actin oxidation vary with the oxidative modification and the cell type [45]. In cardiomyocytes, S-nitrosylation of α-actin correlates with enhanced relaxation and impaired contraction [46]. In endothelial cells, oxidation of actin seems to be essential for proper cell migration [47]. Oxidative modification of actin may sensitize smooth muscle cells to contractile stimuli and contribute to overactive detrusor contractions in bladder ischemia (Figure 6). PTMs by means of redox mechanisms may compromise the dynamic properties of actin and alter actin regulatory proteins and signaling components. In addition, oxidation may provoke actin depolymerization by redox enzymes and post-translationally modify actin reaction to contractile stimuli.

The reactome pathway analysis of the ischemia-associated ncAA-containing proteins also showed that bladder ischemia provoked differential (post-translational) modifications of the proteins involved in HSF1-dependent transactivation pathway, including crystallin, heat shock protein 90 alpha family class B member 1, heat shock protein 90 alpha class A member 1 and heat shock protein family A (HSP70) member 8, suggesting a cellular stress response to the compromised energy homeostasis conditions. HSF1-dependent transactivation plays an important role in ischemia-regulated cellular processes. HSF1 upregulates the expression of heat shock proteins (HSP) under stress conditions to strengthen the cellular defensive capacity to protect cells against stress-mediated injuries [48]. HSF1 protects mitochondrial integrity by regulating caspase activators and inhibiting mitochondrial apoptotic pathways. Analysis of heart tissue specimens from animal models suggests that myocardial apoptosis induced by ischemia-reperfusion is reduced in HSF1 transgenic mice in comparison with wild-type control mice [48]. HSF1 can also protect vascular endothelial cells from oxidative stress-induced apoptosis.

In addition, the reactome pathway analysis of the ischemia-associated ncAA-containing proteins indicated that ischemia provoked differential (post-translational) modifications of the bladder proteins involved in tetrahydrobiopterin (BH4) synthesis, recycling, salvage and regulation. BH4 is a coenzyme of aromatic amino acid hydroxylases and an important cofactor for numerous enzymes, including those involved in nitric oxide synthesis. The pathway of BH4 synthesis, recycling, salvage and regulation is closely related to the pathogenesis of hypertension, ischemia/reperfusion injury and hypertrophy. In ischemic disorders, BH4 levels decrease because of its oxidation and/or reduced biosynthesis, which can lead to functional uncoupling of NOS and impairment of the NO pathway. Under ischemic conditions, oxidative stress increases because oxidized BH4 augments superoxide anion production and increases the levels of peroxynitrite, leading to further BH4 oxidation. Impairment of BH4 represents an important cellular defect involved in the dysfunction of the endothelium and underlying muscle following ischemia/reperfusion injury. The protective roles of BH4 in ischemia and under ischemia/reperfusion conditions appear to involve mechanisms beyond its intrinsic radical scavenging activities. These findings support a potential beneficial role of BH4 against ischemic damage in conditions involving free radical injury, cellular stress and stress response molecules.

In order to elucidate the physiological consequences of ischemia-associated protein modifications, we carried out comprehensive functional analyses using the PANTHER and STRING bioinformatics tools. Assessment of protein class with PANTHER revealed that cytoskeletal proteins comprised the largest group among the ischemia-associated ncAA-containing proteins. Protein–protein interaction network analysis by STRING implied that between the upregulated ischemia-associated ncAA-containing proteins, the protein–protein interaction networks were broadly divided into three functional clusters: cytoskeleton-associated proteins, cell signaling-related proteins and binding/transport-related proteins. Protein–protein interaction network analysis using STRING suggested that interaction networks between the downregulated ischemia-associated ncAA-containing proteins were also broadly divided into three clusters: molecular chaperones, metabolic enzymes and cytoskeleton-associated proteins. Among the molecular chaperones cluster, stress responsive heat shock proteins (HSPs) were predominant, which might be involved in the regulation of the apoptosis signaling pathway, epidermal growth factor (EGF) receptor signaling pathway, gonadotropin-releasing hormone receptor pathway and vascular endothelial growth factor (VEGF) signaling pathway. One of the mechanisms that trigger cellular strain after stress, such as that induced by ischemia, is protein damage and misfolding [49]. Stressed cells upregulate HSPs as a defense mechanism to maintain cell function by restoring cellular homeostasis. HSPs’ biological function, as molecular chaperones, is to either repair denatured proteins or promote their degradation [50,51]. PTMs of HSP90, the most widely studied HSP, alter its ATPase activity, co-chaperone and client protein binding, client protein maturation, HSP90 subcellular localization and degradation, and HSP90 inhibitor sensitivity [52,53,54]. Through effects on the stability and activity of HSP90 client proteins, HSP90 modifications affect downstream cellular processes, including cell cycle and proliferation, cytoskeleton remodeling and migration, transcription, angiogenesis and tumor formation, DNA repair, apoptosis and metabolism [55]. HSP90 regulates proper protein folding and promotes stabilization of proteins under cellular stress conditions. PTMs of HSP90 destabilize proteins, disrupt their proper folding and compromise cellular protective mechanisms in the presence of stress conditions. Downstream impacts of HSP90 modifications include the disruption of cellular processes including DNA repair and cell cycle control. These changes may provoke cellular stress responses and contribute to cell danger signaling in bladder ischemia (Figure 6).

Among the metabolic enzymes cluster, significantly differential modifications of the ATP synthase subunit alpha (Atp5a1) and ATP synthase subunit beta (Atp5b) in ischemia may have significant functional implications. Cellular structural damage under ischemic conditions involves mitochondrial degradation and a significant decrease in mitochondrial ATP synthesis. Lack of ATP leads to depolarization of the plasma membrane and activation of voltage-gated calcium channels, allowing the influx of calcium into the cells. Increased intracellular calcium contributes to smooth muscle contraction and the formation of reactive oxygen species, ultimately leading to oxidative injury and cell death. Bladder ischemia involves the disruption of cell-to-cell junctions and subsequent reorganization of the cytoskeleton. Analysis of protein class revealed that cytoskeletal proteins comprised the largest group among the ischemia-associated ncAA-containing proteins and protein–protein interaction networks showed that the cytoskeleton-associated protein cluster was present in both upregulated and downregulated ncAA-containing proteins in ischemia. Mitochondrial injury and ATP depletion due to chronic ischemia provoke organelle reactions and compromise cytoskeleton structural integrity [56].

In summary, our study represents the first global assessment of ischemic bladder protein modifications in a rat model. Using a systematic proteomic approach, we found the widespread formation of non-coded amino acids (ncAAs) in bladder ischemia, which seemed to involve post-translational protein modifications and amino acid substitution mechanisms. Pathway, gene ontology and protein–protein interaction network analyses of the ischemia-associated ncAA-containing proteins indicated that ischemia provoked differential (post-translational) modifications of the contractile proteins and stress response molecules in the bladder. These modifications may imply dysregulation of downstream pathways, with significant structural and functional consequences. These findings may provide the foundation for future research regarding the validation and clinical translation of the identified biomarkers in bladder dysfunction. The proteomic approach could be applied to a variety of tissues from ischemic organs in order to systematically identify disease-associated ncAAs as potential diagnostic biomarkers and therapeutic targets.

## Figures and Tables

**Table 1:** Ischemia-associated delta mass clusters (R^2^ > 0.5, ratio > 2-fold, *p* < 0.05).

^a^ Delta Mass Clusters (Da)	Calculated Mass (Da)	^b^ Error	^c^ Possible Chemistry	^d^ Freq	Counts
14.0172	14.0156	0.0016	Methylation, D > E, N > Q, G > A, S > T, V > I/L	2	418
12.0014	12.0000	0.0014	+Carbon	2	47
183.0368	183.0354	0.0014	Aminoethylbenzene-sulfonylation	5	45
−33.9858	−33.9844/−33.9877	0.0014/0.0019	F > L/I, C > deHA, M > P	5	33
27.9961	27.9949	0.0012	Formylation, S > D, T > E	6	31
156.1029	156.1011	0.0018	Arg addition	4	20
−17.0252	−17.0265	0.0014	Deamination, pyroglutamate	2	15
305.0703	305.0682	0.0021	glutathione disulfide	1	15
42.0117	42.0106	0.0012	Acetylation, S > E	1	6
31.9913	31.9898	0.0015	Dioxidation, P > E	1	5
−2.0141	−2.0157	0.0015	Disulfidelation, V > P, −2H(didehydro)	1	4
43.9912	43.9898	0.0013	Carboxylation, A > D	1	3

The 12 delta mass clusters were significantly dysregulated in ischemic tissues (R^2^ > 0.5, ratio > 2-fold, *p* < 0.05), which were considered as potential ischemia-associated protein modifications. Note: ^a^ Delta Mass Clusters: the mass difference between the coded amino acids and the actual protein residues; ^b^ Error: the mass difference between Delta Mass Cluster (Da) and Calculated Mass (Da); ^c^ Possible Chemistry: the possible post-translational modification for each delta mass, “>”represents 20 amino acid substitutions; ^d^ Freq (frequency) is the number of ncAAs which had each delta mass.

**Table 2:** Ischemia-associated ncAA-containing proteins (R^2^ > 0.5, ratio > 2-fold, *p* < 0.05).

Upregulated ncAA-Containing Proteins		Downregulated ncAA-Containing Proteins	
Protein Name	Protein ID	Protein Name	Protein ID
Hemoglobin subunit alpha-1/2	P01946	Heat shock 70 kDa protein 1A	P0DMW0
Actin, cytoplasmic 2	P63259	Oligoribonuclease, mitochondrial	Q5U1X1
Protein phosphatase 1 regulatory subunit 12A	Q10728	Phosphoglycerate mutase 1	P25113
Serum albumin	P02770	Serum albumin	P02770
Tropomyosin beta chain	P58775	Serine protease inhibitor A3K	P05545
Actin, aortic smooth muscle	P62738	Desmin	P48675
Desmin	P48675	Collagen alpha-2(I) chain	P02466
Alpha-actinin-1	Q9Z1P2	Transgelin	P31232
Cysteine and glycine-rich protein 1	P47875	L-lactate dehydrogenase A chain	P04642
Prothymosin alpha	P06302	Serpin H1	P29457
Complement component C9	Q62930	Alpha-1-antiproteinase	P17475
Glutathione S-transferase P	P04906	Creatine kinase B-type	P07335
Hemoglobin subunit beta-1	P02091	Ubiquitin carboxyl-terminal hydrolase isozyme L1	Q00981
Histone H1.0	P43278	Alpha-crystallin B chain	P23928
Calmodulin-1	P0DP29	ATP synthase subunit beta, mitochondrial	P10719
14-3-3 protein zeta/delta	P63102	Elongation factor 1-delta	Q68FR9
Glyceraldehyde-3-phosphate dehydrogenase	P04797	40S ribosomal protein S4, X isoform	P62703
Alpha-1-inhibitor 3	P14046	Lactoylglutathione lyase	Q6P7Q4
Non-muscle caldesmon	Q62736	ATP synthase subunit alpha, mitochondrial	P15999
Calponin-1	Q08290	Sepiapterin reductase	P18297
Polymerase I and transcript release factor	P85125	Ig gamma-2B chain C region	P20761
Transgelin	P31232	Myosin regulatory light polypeptide 9	Q64122
Alpha-enolase	P04764	Four and a half LIM domains protein 1	Q9WUH4
Histone H1.4	P15865	Myosin light polypeptide 6	Q64119
T-kininogen 2	P08932	Gelsolin	Q68FP1
Cytochrome b5	P00173	14-3-3 protein zeta/delta	P63102
Tubulin alpha-1B chain	Q6P9V9	Non-muscle caldesmon	Q62736
Caveolin-1	P41350	Selenium-binding protein 1	Q8VIF7
Major urinary protein	P02761	Heat shock protein HSP 90-alpha	P82995
Heat shock protein HSP 90-beta	P34058	Hemoglobin subunit beta-2	P11517
		Heat shock protein beta-6	P97541
		Cofilin-1	P45592
		Heat shock cognate 71 kDa protein	P63018

**Table 3:** Reactome pathway analysis.

Function	Count	*p*-Value	Protein Name
Smooth Muscle Contraction	5	1.4 × 10^−5^	ACTA2, MYL9, CALD1, CALM1, TPM2
Scavenging of heme from plasma	4	9.4 × 10^−5^	ALB, HBB2, HBB1, HBA1
HSF1-dependent transactivation	4	1.1 × 10^−4^	CRYAB, HSP90AB1, HSP90AA1, HSPA8
Tetrahydrobiopterin (BH4) synthesis, recycling, salvage and regulation	3	1.9 × 10^−3^	CALM1, HSP90AA1, SPR
Attenuation phase	3	1.9 × 10^−3^	HSP90AB1, HSP90AA1, HSPA8
Erythrocytes take up oxygen and release carbon dioxide	3	2.2 × 10^−3^	HBB2, HBB1, HBA1
eNOS activation	3	2.2 × 10^−3^	CALM1, CAV1, HSP90AA1

The ischemia-associated non-coded amino acid (ncAA)-containing proteins (R^2^ > 0.5, ratio > 2-fold, *p* < 0.05), defined by pathway analysis using DAVID 6.8. The top 7 pathways are shown.

## Data Availability

Data is contained within this article or Supplementary Materials.

## Supplementary Materials

The following are available online at https://www.mdpi.com/article/10.3390/cells10051031/s1, Table S1: Non-coded amino acid (ncAA)-containing protein sites in rat bladder (R^2^ > 0.5). Table S2: Ischemia-associated delta mass clusters (R^2^ > 0.5, ratio > 2-fold, *p* < 0.05). Table S3: Ischemia-associated ncAA-containing proteins (R^2^ > 0.5, ratio > 2-fold, *p* < 0.05).
